# *Bla*_TEM_-positive *Salmonella enterica* serovars Agona and Derby are prevalent among food-producing animals in Chongqing, China

**DOI:** 10.3389/fmicb.2023.1011719

**Published:** 2023-05-25

**Authors:** Jiacui Lai, Hao Mu, Bingqian Zhou, Jiawei He, Xiangning Cheng, Yujie Gan, Meiyuan Zhao, Mengqi Xie, Yang Zhang, Ying He, Yujiao Yang, Jian Wang, Haoju Wang, Honglei Ding

**Affiliations:** ^1^Laboratory of Veterinary Mycoplasmology, College of Veterinary Medicine, Southwest University, Chongqing, China; ^2^Institute of Veterinary Sciences and Pharmaceuticals, Chongqing Academy of Animal Sciences, Chongqing, China; ^3^Agricultural Service Center, Sub-District of Rongchang, Chongqing, China

**Keywords:** serovar, multidrug resistance, ESBL, PMQR, *bla*_TEM_, *parC*

## Abstract

*Salmonella* is one of the most important foodborne zoonotic pathogens, causing global morbidity and mortality in both humans and animals. Due to the extensive use of antimicrobials in food-producing animals, the antimicrobial resistance of *Salmonella* has attracted increasing attention globally. There have been many reports concerning the antimicrobial resistance of *Salmonella* from food-producing animals, meats and the environment. However, few studies on *Salmonella* from food-producing animals have been reported in Chongqing municipality, China. The aim of the present study was to determine the prevalence, serovar diversity, sequence types, and antimicrobial resistance of *Salmonella* isolated from livestock and poultry in Chongqing. Meanwhile, we also want to know the presence of β-lactamase genes, plasmid-mediated quinolone resistance (PMQR) genes and quinolone resistance-determining region (QRDR) mutations of *Salmonella* isolates. A total of 129 *Salmonella* strains were recovered from 2,500 fecal samples at 41 farms from pigs, goats, beef cattle, rabbits, chickens, and ducks. Fourteen serovars were identified, with *S.* Agona and *S.* Derby being the dominant serovars. The 129 isolates had high resistance to doxycycline (87.6%), ampicillin (80.6%), tetracycline (79.8%), trimethoprim (77.5%), florfenicol (76.7%) chloramphenicol (72.9%), and trimethoprim-sulfamethoxazole (71.3%), but were susceptible to cefepime. A total of 114 (88.4%) isolates showed multidrug resistant phenotypes. The prevalence of β-lactamase genes in *Salmonella* isolates was 89.9% (116/129), and among these isolates, 107 (82.9%) harbored *bla*_TEM_, followed by *bla*_OXA_ (26, 20.2%), *bla*_CTX-M_ (8, 6.2%), and *bla*_CMY_ (3, 2.3%). In addition, *qnrB*, *qnrD*, *qnrS*, *oqxA*, *oqxB*, and *aac(6′)-Ib-cr* were detected in 11, 2, 34, 34, 43, and 72 PMQR-producing isolates, respectively. Moreover, QRDR mutations were very common in PMQR-positive *Salmonella* isolates (97.2%, 70/72) with mutation(s) in *parC* or combinative mutations in *gyrA* and *parC*. More significantly, 32 extended spectrum beta-lactamase (ESBL)-producing isolates were identified, and 62.5% of them were found to harbor one to four PMQR genes. Furthermore, 11 sequence types were identified from the isolates, and most of ESBL-producing isolates were attributed to ST34 (15.6%) and ST40 (62.5%). The coexistence of PMQR genes with β-lactamase genes and the extensive mutations in QRDR present in *Salmonella* isolates from food-producing animals suggest a potential threat to public health. Reasonable utilization and strict control strategies for antimicrobials in animal husbandry and animal treatment are necessary to reduce the emergence and dissemination of drug-resistant *Salmonella* isolates.

## 1. Introduction

*Salmonella* is considered one of the most important foodborne pathogens causing global morbidity and mortality in both humans and animals (Nichols et al., 2021; Harrison et al., 2022; Li et al., 2022). According to data from the World Health Organization, nontyphoidal *Salmonella* infection causes 155,000 deaths worldwide, and approximately 93.8 million gastroenteritis cases are closely related to nontyphoidal *Salmonella* infection per year (Majowicz et al., 2010). According to unpublished data from Chinese Center for Disease Control and Prevention surveillance system, 549 out of 100,000 people carried *Salmonella* in 2013. As one of the most important carriers, food-producing animals can transfer *Salmonella* to humans through the food chain (Foley and Lynne, 2008; Marus et al., 2019).

Adding antimicrobials to feed and drinking water has long been an important means for farms to prevent and treat bacterial diseases, including salmonellosis. However, under long-term exposure to antimicrobials, bacteria have developed resistance to antimicrobials and even multidrug resistance (MDR), which has become a serious challenge to public health (Nichols et al., 2022). Extended-spectrum cephalosporins and quinolones are two classes of antimicrobials often used at farms, where resistance to *Salmonella* is emerging due to the misuse and overuse of antimicrobials. Resistance to cephalosporins is primarily due to the acquisition of β-lactamases that are mainly carried by transferable plasmids and transposons (Ghafourian et al., 2015). The major mechanisms of quinolone resistance have been elucidated to be chromosomal mutations in the quinolone resistance-determining regions (QRDRs) and the presence of plasmid-mediated quinolone resistance (PMQR) genes, which might aid the selection and facilitate the mutation of fluoroquinolone resistance genes (Kuang et al., 2018a). There have been numerous reports of antimicrobial resistance (AMR), a major global public health concern that has the potential to destroy the effectiveness of antimicrobials, and resistance genes about *Salmonella* around the world (Hetman et al., 2022; Canning et al., 2023; Igbinosa et al., 2023). In China, there are also many reports of AMR in *Salmonella* from food-producing animals and their products, such as pigs, poultry and cattle, involving many provinces (Tang et al., 2022; Wang et al., 2023). Moreover, many of these *Salmonella* isolates were resistant to β-lactamases and quinolone, and harbor β-lactam resistance genes and PMQR genes, as well as the chromosomal mutations in the QRDRs. However, there are few reports of AMR and resistance genes of *Salmonella* from food-producing animals in Chongqing, a province-level municipality.

In this study, we investigated *Salmonella* strains isolated from food-producing animal feces and analyzed their serovar diversity, sequence types, and AMR. Furthermore, we compared the relationship between phenotypes and genotypes regarding the resistance towards β-lactams and quinolones.

## 2. Materials and methods

### 2.1. Isolation and identification of *Salmonella*

A total of 2,500 fecal samples were collected from 1,600 pigs, 400 goats, 100 beef cattle, 50 rabbits, 300 chickens, and 50 ducks between September 2016 and May 2019 (Supplementary Table S1). Since numbers of rabbits and ducks were far lower than those of pigs and chickens in Chongqing, to avoid repetitive clones, we collected 50 fecal samples each from rabbits and ducks. Approximately 1 g of each sample was pre-enriched in 10 mL of sterile buffered peptone water (BPW) at 37°C for 8 h. Then, 0.1 mL of the suspension was added to 5 mL of tetrathionate broth (TTB) and incubated at 42°C for 18–24 h. After selective enrichment, the suspensions were streaked onto xylose lysine tergitol 4 (XLT-4) plates and incubated at 37°C for 18–48 h. Five suspected colonies were picked from each plate and confirmed by amplifying the *invA* gene, which is specific for *Salmonella*. Primers designed by our group were invA-F (5’-GAAATTATCGCCACGTTCGGGCA-3′) and invA-R (5’-TCATCGCACCGTCAAAGGA-3′). Finally, one confirmed clone was selected randomly, even if more than one clone was identified as *Salmonella* at the same plate. All identified isolates were aliquoted and stored at −80°C in Luria-Bertani (LB) broth containing 50% glycerol (Cody et al., 2008).

### 2.2. *Salmonella* serotyping

*Salmonella* isolates were serotyped using slide agglutination with hyperimmune sera (Tianrun, Ningbo, China), and the results were interpreted according to the Kauffmann-White scheme.

### 2.3. Multilocus sequence typing

Seven housekeeping genes (*aroC*, *dnaN*, *hemD*, *hisD*, *purE*, *sucA*, and *thrA*) were selected to carry out MLST. Primers (Supplementary Table S2) were synthetized according to the published data (General Administration of Quality Supervision, Inspection and Quarantine of the People’s Republic of China, 2017) and the sequence type (ST) was assigned according to the MLST database.^1^

### 2.4. Antimicrobial susceptibility testing

The antimicrobial susceptibility of *Salmonella* isolates was determined using the disk diffusion method on Mueller-Hinton agar plates and broth dilution method with cation-adjusted Mueller-Hinton broth according to the guidelines of Clinical and Laboratory Standards Institute standards CLSI M100-S32 (CLSI, 2022) and VET08Ed4E (CLSI, 2019). Twenty-seven antimicrobials were tested by the disk diffusion method: ampicillin (AMP), cephalexin (LEX), cefazolin (CFZ), cefoxitin (FOX), cefotaxime (CTX), ceftriaxone (CRO), ceftazidime (CAZ), cefepime (FEP), imipenem (IPM), aztreonam (ATM), streptomycin (STR), kanamycin (KAN), gentamicin (GEN), amikacin (AMK), tetracycline (TET), doxycycline (DOX), chloramphenicol (CHL), florfenicol (FFC), nalidixic acid (NAL), norfloxacin (NOR), ciprofloxacin (CIP), enrofloxacin (ENO), ofloxacin (OFX), enoxacin (ENX), gatifloxacin (GAT), trimethoprim-sulfamethoxazole (SXT), and trimethoprim (TMP). However, the broth dilution method tested 26 antimicrobials, except for enrofloxacin, for there was no interpretive criterion of this drug by using broth dilution method. The results were interpreted according to the standards described by CLSI M100-S32 (CLSI, 2022) and VET08Ed4E (CLSI, 2019). *Escherichia coli* ATCC®* 25922 was used as the quality control.

### 2.5. Detection of β-lactamase genes, PMQR genes and mutations within the QRDR

To analyze the resistance mechanisms, we detected nine β-lactamase genes, 10 PMQR genes and identified mutations in the QRDRs of DNA gyrase and topoisomerase IV by means of PCR and sequencing. The β-lactamase genes were *bla*_TEM_, *bla*_CTX-M_, *bla*_SHV_ (Chen et al., 2004), *bla*_OXA_, *bla*_CMY_, *bla*_PSE_ (Qiao et al., 2017), *bla*_PER_ (Qiao et al., 2017), *bla*_VEB_ (Dallenne et al., 2010), and *bla*_GES_ (Dallenne et al., 2010), while the PMQR genes included *qnrA* (Robicsek et al., 2006), *qnrB*, *qnrC* (Cattoir et al., 2007), *qnrD*, qnr*VC, qnrS*, *aac(6′)-Ib-cr*, *oqxA*, *oqxB*, and *qepA*. Two DNA gyrase genes were *gyrA* (Kim et al., 2016) and *gyrB* (Hansen and Heisig, 2003), and two topoisomerase IV genes comprised *parC* (Kim et al., 2016) and *parE* (Kim et al., 2016). The primers and related parameters were listed in Supplementary Table S2. DNA was extracted using a DNA Extraction Kit (Tiangen Biotech, Beijing, China). Sequences of PCR products were aligned and analyzed using BLAST.^2^ The resulting DNA sequences of all PCR products by amplifying QRDR genes were compared with the *S. typhimurium* LT2 genome as a reference.

### 2.6. Statistical analysis

Statistical analysis was performed using GraphPad Prism (Version 8.0). Differences between proportions were calculated using the Chi-square test. *p* ≤ 0.05 were considered to be statistically significant.

## 3. Results

### 3.1. *Salmonella* prevalence and serovars

In this study, 129 (5.2%) isolates of *Salmonella* were obtained from 2,500 fecal samples, among which 104 (6.5%) were recovered from pigs, 11 (2.8%) from goats, three (3.0%) from beef cattle, two (4.0%) from rabbits, seven (2.3%) from chickens, and two (4.0%) from ducks (Table 1; Supplementary Table S1). Overall, the isolation rate among pigs was higher than that for other sources (*p* < 0.05). Moreover, a significantly higher prevalence of *Salmonella* was detected in pigs than in goats and chickens (*p* < 0.05). However, there was no difference in the isolation rate of strains in different years, even from different sources. Fourteen different serovars were identified from the isolates, and *S.* Derby (56, 43.4%) and *S.* Agona (24, 18.6%) were the two most common serovars (Table 1). The distribution of serovars varied among different sources. *S.* Derby (32, 30.8%) and *S.* Agona (24, 23.1%) were more prevalent than other serovars in pigs. However, most isolates recovered from goats (90.9%, 10/11) and all strains acquired from beef cattle, rabbits, and poultry were *S*. Derby, although the number of these isolates from these animals was much less than that of isolates from pigs.

**Table 1:** Prevalence and serotypes of *Salmonella* isolates from animals in Chongqing, China.

Source of sample	Number of isolates	Serovars	Number of serovars
Pigs	104/1600 (6.5%^a^)	*S*. Agona (24), *S*. Anatum (2), *S*. Bredeney (1), *S*. Derby (32), *S*. Kottbus (4), *S*. London (7), *S*. Manhattan (1), *S*. Mbandaka (1), *S*. Newlands (3), *S. paratyphi* B (4), *S*. Regent (7), *S*. Rissen (8), *S*. Stanley (2), *S. typhimurium* (8)	14
Goats	11/400 (2.8%^b^)	*S*. Derby (10), *S*. Stanley (1)	2
Beef cattle	3/100 (3.0%)	*S*. Derby (3)	1
Rabbits	2/50 (4.0%)	*S*. Derby (2)	1
Chickens	7/300 (2.3%^b^)	*S*. Derby (7)	1
Ducks	2/50 (4.0%)	*S*. Derby (2)	1
Total	129/2500 (5.2%)		14

^a,b^Values with different letter superscripts indicate significant differences (*p* < 0.05).

### 3.2. Antimicrobial susceptibility testing of 129 *Salmonella* isolates

**Figure 1:**
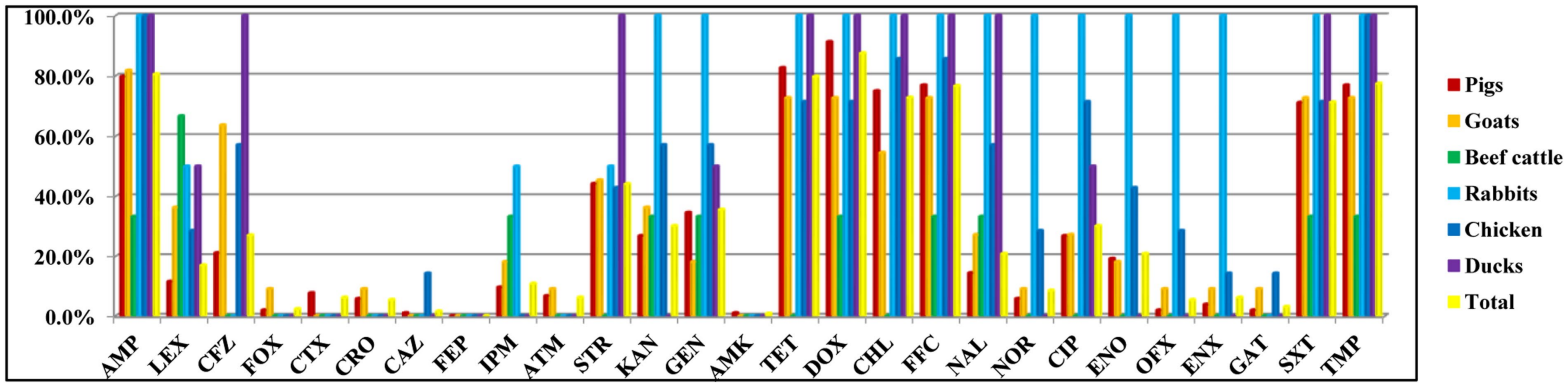
Antimicrobial resistance of *Salmonella* isolated from food-producing animals was determined by disk diffusion method. AMP (ampicillin), LEX (cephalexin), CFZ (cefazolin), FOX (cefoxitin), CTX (cefotaxime), CRO (ceftriaxone), CAZ (ceftazidime), FEP (cefepime), IPM (imipenem), ATM (aztreonam), STR (streptomycin), KAN (kanamycin), GEN (gentamicin), AMK (amikacin), TET (tetracycline), DOX (doxycycline), CHL (chloramphenicol), FFC (florfenicol), NAL (nalidixic acid), NOR (norfloxacin), CIP (ciprofloxacin), ENO (enrofloxacin), OFX (ofloxacin), ENX (enoxacin), GAT (gatifloxacin), SXT (trimethoprim-sulfamethoxazole), and TMP (trimethoprim).

The AMR phenotypes of 129 *Salmonella* isolates exhibited high rates of resistance to doxycycline (87.6%, 113/129), ampicillin (80.6%, 104/129), tetracycline (79.8%, 103/129), trimethoprim (77.5%, 100/129), florfenicol (76.7%, 99/129), chloramphenicol (72.9%, 94/129), and trimethoprim-sulfamethoxazole (71.3%, 92/129). However, all isolates were susceptible to cefepime and less resistant to amikacin (0.8%, 1/129), ceftazidime (1.6%, 2/129), cefoxitin (2.3%, 3/129), gatifloxacin (3.1%, 4/129), ceftriaxone (5.4%, 7/129), ofloxacin (5.4%, 7/129), aztreonam (6.2%, 8/129), and enoxacin (6.2%, 8/129; Figure 1; Table 2; Supplementary Tables S2, S3). Moreover, three (2.3%) isolates were susceptible to all antimicrobial agents, while 126 (97.7%) isolates showed resistance to at least one drug.

**Table 2:** Antimicrobial resistance profiles of *Salmonella* isolates recovered from pigs, goats, beef cattle, rabbits, chickens, and ducks as determined by disk diffusion method.

Antimicrobial	Pigs (*n* = 104)	Goats (*n* = 11)	Beef cattle (*n* = 3)	Rabbits (*n* = 2)	Chickens (*n* = 7)	Ducks (*n* = 2)	Total (*n* = 129)
Ampicillin	83 (79.8)	9 (81.8)	1 (33.3)	2 (100.0)	7 (100.0)	2 (100.0)	104 (80.6)
Cephalexin	12 (11.5)	4 (36.4)	2 (66.7)	1 (50.0)	2 (28.6)	1 (50.0)	22 (17.1)
Cefazolin	22 (21.2)	7 (63.6)	0	0	4 (57.1)	2 (100.0)	35 (27.1)
Cefoxitin	2 (1.9)	1 (9.1)	0	0	0	0	3 (2.3)
Cefotaxime	8 (7.7)	0	0	0	0	0	8 (6.2)
Ceftriaxone	6 (5.8)	1 (9.1)	0	0	0	0	7 (5.4)
Ceftazidime	1 (1.0)	0	0	0	1 (14.3)	0	2 (1.6)
Cefepime	0	0	0	0	0	0	0
Imipenem	10 (9.6)	2 (18.2)	1 (33.3)	1 (50.0)	0	0	14 (10.9)
Aztreonam	7 (6.7)	1 (9.1)	0	0	0	0	8 (6.2)
Streptomycin	46 (44.2)	5 (45.5)	0	1 (50.0)	3 (42.9)	2 (100.0)	57 (44.2)
Kanamycin	28 (26.9)	4 (36.4)	1 (33.3)	2 (100.0)	4 (57.1)	0	39 (30.2)
Gentamicin	36 (34.6)	2 (18.2)	1 (33.3)	2 (100.0)	4 (57.1)	1 (50.0)	46 (35.7)
Amikacin	1 (1.0)	0	0	0	0	0	1 (0.8)
Tetracycline	86 (82.7)	8 (72.7)	0	2 (100.0)	5 (71.4)	2 (100.0)	103 (79.8)
Doxycycline	95 (91.3)	8 (72.7)	1 (33.3)	2 (100.0)	5 (71.4)	2 (100.0)	113 (87.6)
Chloramphenicol	78 (75.0)	6 (54.5)	0	2 (100.0)	6 (85.7)	2 (100.0)	94 (72.9)
Florfenicol	80 (76.9)	8 (72.7)	1 (33.3)	2 (100.0)	6 (85.7)	2 (100.0)	99 (76.7)
Nalidixic acid	15 (14.4)	3 (27.3)	1 (33.3)	2 (100.0)	4 (57.1)	2 (100.0)	27 (20.9)
Norfloxacin	6 (5.8)	1 (9.1)	0	2 (100.0)	2 (28.6)	0	11 (8.5)
Ciprofloxacin	28 (26.9)	3 (27.3)	0	2 (100.0)	5 (71.4)	1 (50.0)	39 (30.2)
Enrofloxacin	20 (19.2)	2 (18.2)	0	2 (100.0)	3 (42.9)	0	27 (20.9)
Ofloxacin	2 (1.9)	1 (9.1)	0	2 (100.0)	2 (28.6)	0	7 (5.4)
Enoxacin	4 (3.8)	1 (9.1)	0	2 (100.0)	1 (14.3)	0	8 (6.2)
Gatifloxacin	2 (1.9)	1 (9.1)	0	0	1 (14.3)	0	4 (3.1)
Trimethoprim-sulfamethoxazole	74 (71.2)	8 (72.7)	1 (33.3)	2 (100.0)	5 (71.4)	2 (100.0)	92 (71.3)
Trimethoprim	80 (76.9)	8 (72.7)	1 (33.3)	2 (100.0)	7 (100.0)	2 (100.0)	100 (77.5)

Unit: %.

A total of 114 (88.4%) isolates exhibited MDR, which was defined as resistant to three or more classes of antimicrobials (Table 3). All strains belonging to *S.* Derby obtained from rabbits and poultry showed MDR profiles. A higher prevalence of MDR isolates from pigs was found among *S.* Derby (93.8%, 30/32), compared with *S.* Agona (75.0%, 18/24), although there was no significant difference. Serovars *S*. Anatum (2), *S*. Bredeney (1), *S*. Kottbus (4), *S*. London (6), *S*. Manhattan (1), *S*. Mbandaka (1), *S*. Newlands (3), *S. paratyphi* B (4), *S*. Regent (7), *S*. Rissen (7), *S*. Stanley (1), and *S. typhimurium* (8) from pigs were MDR (Table 4).

**Table 3:** Multidrug-resistant (MDR) *Salmonella* isolated from animals in Chongqing.

Source of isolates	Number of isolates to indicated number of antimicrobial categories (%)							
	0 (%)	1 (%)	2 (%)	3 (%)	4 (%)	5 (%)	6 (%)	Total of MDR (%)
Pigs	2 (1.9)	5 (4.8)	4 (3.8)	14 (13.5)	27 (26.0)	23 (22.1)	29 (27.9)	93 (89.4)
Goats	1 (9.1)	1 (9.1)			5 (45.5)	1 (9.1)	3 (27.3)	9 (81.8)
Beef cattle	1 (33.3)	1 (33.3)				1 (33.3)		1 (33.3)
Rabbits							2 (100.0)	2 (100.0)
Chicken					2 (28.6)	3(42.8)	2 (28.6)	7 (100.0)
Ducks							2 (100.0)	2 (100.0)
Total	4 (3.1)	7 (5.4)	4 (3.1)	14 (10.9)	34 (26.4)	28 (21.7)	38 (29.5)	114 (88.4)

**Table 4:** Distribution of multidrug-resistant *Salmonella* in different serotypes and animals.

Serotype	Pigs (%)	Goats (%)	Beef cattle (%)	Rabbits (%)	Chickens (%)	Ducks (%)	Total (%)
Agona	18 (75.0)						18 (75.0)
Anatum	2 (100.0)						2 (100.0)
Bredeney	1 (100.0)						1 (100.0)
Derby	30 (93.8)	8 (80.0)	1 (33.3)	2 (100.0)	7 (100.0)	2 (100.0)	50 (89.3)
Kottbus	4 (100.0)						4 (100.0)
London	6 (85.7)						6 (85.7)
Manhattan	1 (100.0)						1 (100.0)
Mbandaka	1 (100.0)						1 (100.0)
Newlands	3 (100.0)						3 (100.0)
Paratyphi B	4 (100.0)						4 (100.0)
Regent	7 (100.0)						7 (100.0)
Rissen	7 (87.5)						7 (87.5)
Stanley	1 (50.0)	1 (100.0)					2 (66.7)
Typhimurium	8 (100.0)						8 (100.0)
Total	93 (89.4)	9 (81.8)	1 (33.3)	2 (100.0)	7 (100.0)	2 (100.0)	114 (88.4)

### 3.3. Prevalence of β-lactamase genes in *Salmonella* isolates

The prevalence of β-lactamase genes in *Salmonella* isolates was 89.9% (116/129), and of these, all isolates collected from rabbits and poultry, and 90.4% (94/104) of pig-associated strains harbored at least one β-lactamase gene. Among the isolates, 107 (82.9%) harbored *bla*_TEM_, followed by *bla*_OXA_ (26, 20.2%), *bla*_CTX-M_ (8, 6.2%), and *bla*_CMY_ (3, 2.3%; Table 5). Strains from goats, beef cattle, rabbits and poultry carried *bla*_TEM_ genes and were resistant to at least one β-lactam drug except for 1 strain isolated from beef cattle. The allele of 104 *bla*_TEM_ was TEM-1 (44.2%, 57/129), TEM-1a (12.4%, 16/129), TEM-1b (7.8%, 10/129), TEM-116 (0.8%, 1/129), and TEM-171 (17.8%, 23/129). *bla*_OXA_ was found in pigs, goats, rabbits and chickens, and its genetic genotypes were *bla*_OXA-1_ (76.9%, 20/26) and *bla*_OXA-10_ (23.1%, 6/26). Three *bla*_CMY-116_-harboring strains were detected, and two were isolated from pigs and one from duck. *bla*_CTX-M_ was amplified from eight pig-associated strains, and the genotypes were *bla*_CTX-M-65_. As the predominant serovars, 79.2% (19/24) *S.* Agona and 87.5% (49/56) *S.* Derby harbored *bla*_TEM_, and the proportions of *bla*_TEM_-positive isolates in both serovars were lower than the proportion of *bla*_TEM_-positive isolates in the total number of *Salmonella* isolates. Beta-lactamase genes in isolates carried one to three *bla* genes (Supplementary Table S4). The most prevalent genotype form was TEM-1 (31.0%, 40/129), followed by TEM-1a (11.6%, 15/129), TEM-171 (10.9%, 14/129), TEM-1b (7.8%, 10/129), and TEM-171 + OXA-1 (7.0%, 9.129). Extended spectrum β-lactamases (ESBLs) are defined as enzymes produced by certain bacteria that are able to hydrolyze β-lactam ring of broad-spectrum β-lactams such as oxyimino-cephalosporins including cefotaxime, ceftriaxone, and ceftazidime (Kawamura et al., 2017). The detection result showed that six isolates carrying *bla*_CTX-M-65_ gene were resistant to the third-generation cephalosporins cefotaxime and ceftriaxone.

**Table 5:** The prevalence of β-lactamase genes in *Salmonella* isolates originating from different animals.

Genotype of β-lactamase gene		Pigs (%)	Goats (%)	Beef cattle (%)	Rabbits (%)	Chickens (%)	Ducks (%)	Total (%)
TEM	TEM-1	44 (42.3)	5 (45.5)	1 (33.3)		5 (71.4)	2 (100.0)	57 (44.2)
	TEM-1a	16 (15.4)						16 (12.4)
	TEM-1b	10 (9.6)						10 (7.8)
	TEM-116	1 (1.0)						1 (0.8)
	TEM-171	17 (16.3)	3 (27.3)	1 (33.3)	1 (50.0)	1 (14.3)		23 (17.8)
OXA	OXA-1	13 (12.5)	3 (27.3)		2 (100.0)	2 (28.6)		20 (15.5)
	OXA-10	6 (5.8)						6 (4.7)
CMY	CMY-116	2 (1.9)					1 (50.0)	3 (2.3)
CTX-M	CTX-M-65	8 (7.7)						8 (6.2)

### 3.4. Distribution of PMQR and QRDR mutations among strains

Fifty-seven strains were resistant to at least one tested quinolone, and most of these strains harbored PMQR gene(s) (82.5%, 47/57) and/or exhibited mutation(s) in QRDR gene(s) (98.2%, 56/57). However, PMQR gene(s) and QRDR point mutation(s) were detected in 82 (63.6%) and 121 (93.8%) *Salmonella* isolates, regardless of whether they were resistant to the quinolones tested. Only *qnrB*, *qnrD*, *qnrS*, *oqxA*, *oqxB*, and *aac(6′)-Ib-cr* were detected in 13.4% (11), 2.4% (2), 41.5% (34), 41.5% (34), 52.4% (43), and 50.0% (41) of the PMQR-producing isolates, respectively (Table 6). *qnrA*, *qnrC*, *qnrVC*, and *qepA* were not present in any isolate. The genotypes of *qnrB*, *qnrD*, *oqxA*, and *oqxB* were *qnrB6*, *qnrD1*, *oqxA1*, and *oqxB5*. Nevertheless, *qnrS* represented *qnrS1* (6.1%, 5/82), *qnrS2* (13.4%, 11/82) and *qnrS10* (22.0%, 18/82). Similar to β-lactamase gene-containing isolates, there were 20 genotype forms of PMQR genes in 82 PMQR-positive strains (Supplementary Table S5). There were 14 and six isolates carrying a single PMQR gene, *qnrS10* and *oqxB5*, respectively. Moreover, 10 isolates carried the *oqxA1* and *oqxB5* genes, and eight isolates carried *qnrS2*, *oqxA1*, *oqxB5*, and *aac(6′)-Ib-cr* simultaneously. Interestingly, most *qnrS2*-positive strains (90.9%, 10/11) were positive for *aac(6′)-Ib-cr*.

**Table 6:** The prevalence of PMQR genes in *Salmonella* isolates originating from different animals.

Genotype of PMQR genes		Pigs (%)	Goats (%)	Rabbits (%)	Chickens (%)	Ducks (%)	Total (%)
*qnrB*	*qnrB6*	11 (10.6)					11 (8.5)
*qnrD*	*qnrD1*	2 (1.9)					2 (1.6)
*qnrS*	*qnrS1*	3 (2.9)	2 (18.2)				5 (3.9)
	*qnrS2*	7 (6.7)	3 (27.3)	1 (50.0)			11 (8.5)
	*qnrS10*	16 (15.4)	1 (9.1)		1 (14.3)		18 (14.0)
*oqxA*	*oqxA1*	28 (26.9)	2 (18.2)	2 (100.0)	2 (28.6)		34 (26.4)
*oqxB*	*oqxB5*	39 (37.5)	2 (18.2)	1 (50.0)	1 (14.3)		43 (33.3)
*aac (6′)-Ib-cr*		31 (29.8)	3 (27.3)	2 (100.0)	4 (57.1)	1 (50.0)	41 (31.8)

QRDR mutations were very common in PMQR-positive *Salmonella* isolates (97.5%, 80/82; Table 7). A single mutation in *parC* (T57S) was detected in isolates obtained from goats (6), rabbits (2) and chickens (5), and 91.2% (62) pig-associated PMQR-positive isolates. Double mutations and triple mutations in *parC* were found in two (S57T/G72C and S57T/L131M) and one (S57T/G72C/L131M) isolates, respectively. A combination of mutations in *gyrA* (V143G) and in *parC* (S57T) was found in two isolates. However, both mutations in *gyrA* (S83L) and *parC* (S57T) were detected in one isolate.

**Table 7:** Distribution of QRDR mutations in PMQR-positive *Salmonella* isolates.

QRDR mutation(s)						
*gyrA*	*parC*	Pigs	Goats	Rabbits	Chickens	Total
None	S57T	62	6	2	5	75
None	S57T, G72C	1				1
None	S57T, L131M	1				1
None	S57T, G72C, L131M	1				1
V143G	S57T	2				2

### 3.5. Mechanisms of ciprofloxacin resistance among the isolates

To detect the main mechanism for the presence of 39 CIP-resistant isolates, we found that the most frequent mutation in *parC* was S57T (89.7%, 35/39; Table 8). Moreover, double mutations in *gyrA* and *parC* of three isolates mentioned above were also resistant to ciprofloxacin. Most of CIP-resistant isolates harbored at least one PMQR gene, except for three strains. Surprisingly, one isolate, resistant to ciprofloxacin, only contained *aac(6′)-Ib-cr* and no mutation was identified at any QRDR genes.

**Table 8:** Distribution of QRDR mutations and PMQR genes among the 39 ciprofloxacin-resistant *Salmonella* isolates.

QRDR mutations	PMQR genes	No. of isolates (%)
*parC* (S57T)		2
	*qnrB6* + *oqxA1* + *oqxB5* + *aac(6′)-Ib-cr*	3
	*qnrB6* + *aac(6′)-Ib-cr*	2
	*qnrD1* + *oqxA1* + *oqxB5* + *aac(6′)-Ib-cr*	1
	*qnrS1*	1
	*qnrS1* + *oqxB5*	1
	*qnrS2*	1
	*qnrS2* + *oqxA1* + *oqxB5* + *aac(6′)-Ib-cr*	5
	*qnrS2* + *aac(6′)-Ib-cr*	1
	*qnrS10*	1
	*qnrS10* + *oqxA1* + *oqxB5*	1
	*oqxA1* + *oqxB5*	3
	*oqxA1* + *oqxB5* + *aac(6′)-Ib-cr*	4
	*oqxA1* + *aac(6′)-Ib-cr*	2
	*oqxB5*	1
	*oqxB5* + *aac(6′)-Ib-cr*	2
	*aac(6′)-Ib-cr*	4
*gyrA* (S83L) and *parC* (S57T)		1
*gyrA* (V143G) and *parC* (S57T)	*qnrS2* + *oqxA1* + *oqxB5* + *aac(6′)-Ib-cr*	1
	*qnrS2* + *aac (6′)-Ib-cr*	1
	*aac(6′)-Ib-cr*	1
Total		39

### 3.6. Characterization of PMQR genes in ESBL-producing isolates

Thirty-two ESBL-producing strains were identified from the isolates, and each strain carried one of ESBL genes from three genotypes, namely *bla*_TEM-116_, *bla*_TEM-171_, and *bla*_CTX-M-65_. The distribution of PMQR-encoding genes among 32 ESBL-producing *Salmonella* isolates was shown in Table 9. Twenty (62.5%) ESBL-producing *Salmonella* isolates were found to harbor one to four PMQR genes, and combinations of *qnrS2* + *oqxA1* + *oqxB5* + *aac(6′)-Ib-cr* (30%, 6/20) was the most common combination type, followed by *oqxA1* + *oqxB5* (25.0%, 5/20).

**Table 9:** Distribution of PMQR genes among ESBL-producing *Salmonella* isolates.

ESBL gene	PMQR genes	No. of isolates
*bla*_TEM-116_	*oqxB5*	1
*bla*_TEM-171_	*qnrS1*	1
	*qnrS2* + *oqxA1* + *oqxB5* + *aac(6′)-Ib-cr*	6
	*oqxA1* + *oqxB5*	3
	*oqxA1* + *oqxB5* + *aac(6′)-Ib-cr*	1
	*oqxA1* + *aac(6′)-Ib-cr*	1
	*oqxB5*	1
	*aac(6′)-Ib-cr*	3
		7
*bla*_CTX-M-65_	*oqxA1*	1
	*oqxA1* + *oqxB5*	2
		5
Total		32

### 3.7. Sequence types and the relatedness with ESBL-producing isolates

In total, 11 sequence types were identified from the *Salmonella* isolates, including ST11, ST19, ST34, ST40, ST155, ST279, ST413, ST463, ST469, ST1498, and ST1499 (Table 10). ST34 (10.9%, 14/129), ST40 (36.4%, 47/129), ST463 (12.4%, 16/129), and ST469 (14.7%, 19/129) were the more popular sequence types. Most of isolates containing *bla*_TEM-171_ (69.6%, 16/23) and the *bla*_TEM-116_-positive isolate were identified as ST40. However, isolates harboring *bla*_CTX-M-65_ were attributed to ST34 (62.5%, 5/8) and ST40 (37.5%, 3/8).

**Table 10:** Sequence types of *Salmonella* isolates, especially the ESBL-producing strains.

Sequence type	No. of isolates (%)	*bla*_TEM-116_-positive (%)	*bla*_TEM-171_ positive (%)	*bla*_CTX-M-65_ positive (%)
ST11	2 (1.6)			
ST19	2 (1.6)			
ST34	14 (10.9)		2 (8.7)	5 (62.5)
ST40	47 (36.4)	1 (100.0)	16 (69.6)	3 (37.5)
ST155	13 (10.1)		1 (4.3)	
ST279	11 (8.5)		2 (8.7)	
ST413	1 (0.8)		1 (4.3)	
ST463	16 (12.4)			
ST469	19 (14.7)			
ST1498	2 (1.6)		1 (4.3)	
ST1499	2 (1.6)			
Total	129	1	23	8

## 4. Discussion

As major reservoirs of bacteria and an important part of food chain, food-producing animals play an irreplaceable role in the transmission of *Salmonella*. It is of great significance for the prevention and control of salmonellosis in humans and animals to monitor the prevalence and dynamics of antimicrobial resistance and resistant genes of *Salmonella* originating from food-producing animals in countries or regions with extensive livestock and/or poultry breeding. In this study, we investigated the prevalence, serovar distribution, sequence types, antimicrobial susceptibility phenotypes, the emergence of β-lactamase and PMQR genes, and the occurrence of mutations in the QRDR of *Salmonella* isolates acquired from pigs, goats, beef cattle, rabbits, and poultry in Chongqing, China.

The number of samples collected from different animals was roughly consistent with the breeding density of different animals in Chongqing, and the number of samples collected from pigs (1600) and chickens (300) was much higher than those from beef cattle (100), rabbits (50) and ducks (50). Overall, 104 *Salmonella* isolates were recovered from 1,600 fecal samples of pigs, showing a prevalence of 6.5%, higher than the prevalence in Italy (3.4%; Siddi et al., 2021) but much lower than the prevalence in England (19.5%; Wales et al., 2013) and Thailand (37.54%; Phongaran et al., 2019). Moreover, the prevalence of *Salmonella* in pigs was lower than the prevalence in Shanghai (26.3%; Tian et al., 2021), Shandong (11.1%; Zhao et al., 2017) and other Chinese provinces (Li et al., 2013; Zhang et al., 2016; Kuang et al., 2018b; Zhang et al., 2019). Similarly to the prevalence of *Salmonella* from pigs, the prevalence of *Salmonella* isolated from chickens in Chongqing is lower than that of *Salmonella* from chickens in other countries and regions (Zhao et al., 2017; Caffrey et al., 2021; Sarker et al., 2021), even with a few exceptions (Li et al., 2013).

Previous studies have reported that ducks are important reservoirs of *Salmonella* (Li et al., 2013; Zhao et al., 2017; Zhang et al., 2019; Kim et al., 2021; Kang et al., 2022), and Chinese duck production exceeds 90% of all ducks globally; therefore, we investigated *Salmonella* in duck samples. The results indicate that the isolation rate of *Salmonella* in ducks is lower than in other regions of China (Li et al., 2013; Zhao et al., 2017; Zhang et al., 2019; Kim et al., 2021; Kang et al., 2022). Moreover, we isolated *Salmonella* from herbivores, including goats, beef cattle, and rabbits, with isolation rates of 2.8, 3.0 and 4.0%, respectively. Compared with other countries, the isolation rate of *Salmonella* in beef cattle and goats is low (Bosilevac et al., 2015; Thomas et al., 2020; Gutema et al., 2021). Such differences in isolation rates among different animals in our study and other reports can be interpreted based on differences in region, animal species, sample types, collection seasons, culture methods, isolation methodologies, culture media, and local environmental conditions (Kuang et al., 2015).

Among the strains isolated from pigs, 12 serovars were identified in addition to the dominant serovars Agona and Derby. These, previously reported to having been recovered in pigs, included *S.* Anatum (Li et al., 2013; Siddi et al., 2021), *S.* Bredeney (Grafanakis et al., 2001; Siddi et al., 2021), *S.* Kottbus (Toboldt et al., 2014), *S.* London (Grafanakis et al., 2001; Bonardi et al., 2016), *S.* Manhattan (Wales et al., 2013; Bonardi et al., 2016), *S.* Mbandaka (Tian et al., 2021), *S.* Newlands (Kuang et al., 2015; Li et al., 2019), *S. paratyphi* B (Phongaran et al., 2019), *S.* Rissen (Bonardi et al., 2016; Tian et al., 2021), *S.* Stanley (Kuang et al., 2015; Bonardi et al., 2016) and *S. typhimurium* (Li et al., 2013; Zhao et al., 2017; Tian et al., 2021). However, to the best of our knowledge, this is the first study reporting *S.* Regent recovered from pigs, although this serovar was first reported to be isolated from ducks in Korea (Yoon et al., 2014). This demonstrates a serovar of *Salmonella* that is not common at pig farms in other places, with clearly reported pathogenicity (Duan, 2006). Moreover, there were few reports about *S. paratyphi* B isolated from pigs (Phongaran et al., 2019), and no report about the isolation of *S. paratyphi* B from pigs in China prior to our research. Therefore, monitoring of *S.* Regent and *S. paratyphi* B in pig farms and pork products should be strengthened to prevent potential transmissions to humans through the food chain. In addition, *S.* Derby and *S.* Stanley isolated from goats have not been reported before. The main reason may be that, compared with studies on *Salmonella* obtained from pigs and poultry, there have been fewer studies on goat-associated *Salmonella*.

The resistance of isolates to different antimicrobials was positively correlated with the frequency of antimicrobial use (private communication with farmers or workers). For example, isolates have a high resistance rate to ampicillin and doxycycline, which are often used in the breeding process. However, some antimicrobials are rarely used in farms, but the isolates have high resistance to them. For instance, the use of tetracycline is far lower than the use of doxycycline at farms in China. However, our results show that *Salmonella* isolates had almost identical resistant phenotypes to tetracycline and doxycycline. The reason may be that strains resistant to doxycycline were also resistant to tetracycline because they have the same resistance genes, such as *tet* genes (Pavelquesi et al., 2021). Moreover, the resistant phenotype of isolates to chloramphenicol, which has been banned for more than 20 years in China, was similar to that of florfenicol, which should also be related to the same resistance genes of these two antimicrobials. In general, pig, chicken and rabbit producers use antimicrobials much more frequently than producers of ruminants, reflected in the resistance to antimicrobials of isolates obtained from these two kinds of animals. The resistance rates of *Salmonella* isolated from pigs, chickens and rabbits to most tested antimicrobials were higher than those of *Salmonella* isolated from ruminants. The proportion of multidrug-resistant strains of *Salmonella* isolated from pigs, chickens and rabbits was also higher than that of *Salmonella* strains isolated from ruminants.

In this study, β-lactamase genes were amplified in 89.9% of the strains, of which 82.9% carried the *bla*_TEM_ gene, whose alleles were TEM-1, TEM-1a, TEM-1b, TEM-116, and TEM-171. However, we found that only eight isolates carried *bla*_CTX-M_ gene (6.2%). Most reports on *Salmonella* isolated from food-producing animals, including those in China, and even studies in Sichuan Province adjacent to Chongqing, have shown that *bla*_CTX-M_ was the most widespread ESBL gene. Therefore, the prevalence of *bla*_TEM_ was generally lower than *bla*_CTX-M_ (Zhang et al., 2016; Luk-In et al., 2018; Kuang et al., 2018b; Zhang et al., 2019), although there are a few reports that the prevalence of *bla*_TEM_ was higher than that of *bla*_CTX-M_ (Zhao et al., 2017; Al-Gallas et al., 2022). The prevalence of the β-lactamase gene, especially *bla*_TEM_, is very worrisome. China’s transportation network is very developed, and every village has cement concrete pavements, which makes it very convenient for people to travel but also provides convenience for pathogens to use the same developed transportation network to spread through carriers, such as animals, meat products, contaminated vehicles, and even people. *Salmonella* from food-producing animals is very easy to spread out of Chongqing through the carriers mentioned above. The *bla*_TEM_ gene in the *Salmonella* strains can be transferred into other *bla*_TEM_-negative strains through plasmid conjugation, transformation, transduction, etc., (data not shown), resulting in the widespread prevalence of *bla*_TEM_-positive *Salmonella* outside Chongqing.

The coexistence of PMQR genes in CTX-M-producing isolates has been widely reported worldwide (Li et al., 2014; Zhang et al., 2016). However, there has been no demonstrated linkage between the emergence of *bla*_CTX-M_ and resistance to quinolones, and the emergence of *bla*_CTX-M_ and coexistence of PMQR genes in *Salmonella* isolates. The reason was that all CTX-M-positive isolates were susceptible to seven tested quinolones and 62.5% (5/8) of isolates did not carry any PMQR gene. This indicated that *bla*_CTX-M_ and PMQR are not necessarily cotransferred in some regions or that *bla*_CTX-M_ may be located on chromosomes, such as CTX-M-14 (Zhang et al., 2019; Hamamoto et al., 2020).

The ESBLs phenotype mediates the resistance to third or fourth-generation cephalosporins. In the ESBL-producing isolates, 75% (6/8) of strains carrying *bla*_CTX-M-65_ showed resistance to cefotaxime and ceftriaxone, while only one strain (4.3%, 1/23) harboring *bla*_TEM-171_ exhibited resistance to ceftriaxone. This indicates that *bla*_CTX-M-65_ plays a more important role in resistance to broad-spectrum β-lactams. A research from United States also supported this point (Tate et al., 2017). Therefore, although the proportion of *Salmonella* carrying the *bla*_CTX-M-65_ gene in food-producing animals in Chongqing is low, its resistance to broad-spectrum β-lactams could not be ignored.

The mutations of QRDR genes often occurred in *gyrA*, such as S83Y, S83F, D87G, D87N, and D87Y (Zhang et al., 2016; Wang et al., 2020). The mutations of *parC* were less than the mutations found in *gyrA*, and the amino acid substitution of ParC was usually at the position of 80th (S80R; Zhang et al., 2016; Wang et al., 2020). However, in our study, mutations mainly occurred at the 57th amino acid of the ParC. Similar study has also been reported in *Salmonella* isolated from Guangdong in recent years (Chen et al., 2021), indicating an increasing trend of mutation in the 57th amino acid of the ParC.

The Ministry of Agriculture of China requested that the usage of lomefloxacin, pefloxacin, ofloxacin and norfloxacin in food-producing animals be forbidden beginning December 31, 2016. On October 21, 2021, the Ministry of Agriculture and Rural Affairs of China issued the “national action plan for reducing the use of veterinary antimicrobials (2021–2025).” These actions provided powerful measures for reducing drug-resistant bacteria from food-producing animals. Further longitudinal monitoring of antimicrobial susceptibility, serotypes and resistance genes of *Salmonella* isolates from food-producing animals in the same geographic region should be carried out to compare and evaluate the trends of above parameters.

## 5. Conclusion

In summary, we first analyzed the prevalence, serovar diversity, sequence types, and antimicrobial resistance and examined the β-lactamase, QRDR, and PMQR genes of *Salmonella* strains isolated from livestock and poultry at farms in Chongqing, China. Our findings demonstrated the diversity of serovars of *Salmonella* isolates. Notably, various MDR serovars of *Salmonella* are widespread, which highlights the potential risk of antimicrobial-resistant *Salmonella* foodborne infections. This study emphasizes the significant roles of *bla*_TEM_ genes in β-lactam-resistant isolates and *parC* mutations in quinolone-resistant isolates, especially those that carry PMQR genes.

## Data availability statement

The original contributions presented in the study are included in the article/Supplementary material, further inquiries can be directed to the corresponding author.

## Ethics statement

The animal study was reviewed and approved by IACUC-20190305-01.

## Author contributions

HD, HM, and YH conceived and designed the experiments. JL, HM, BZ, JH, XC, YG, MZ, MX, YZ, and YH performed the experiments. HD and HW analyzed the data. JL, JW, and YY contributed reagents/materials/analysis tools. HD and HM wrote the paper. All authors contributed to the article and approved the submitted version.

## Funding

This work was supported by Chongqing’s Modern Agricultural Industry Technology System Program for Herbivore (2022[12]).

## Conflict of interest

The authors declare that the research was conducted in the absence of any commercial or financial relationships that could be construed as a potential conflict of interest.

## Publisher’s note

All claims expressed in this article are solely those of the authors and do not necessarily represent those of their affiliated organizations, or those of the publisher, the editors and the reviewers. Any product that may be evaluated in this article, or claim that may be made by its manufacturer, is not guaranteed or endorsed by the publisher.

## Footnotes

^1^ https://pubmlst.org/bigsdb?db=pubmlst_salmonella_seqdef

^2^ http://www.ncbi.nlm.nih.gov/BLAST/
