# Supplementary material for: BlaTEM-positive Salmonella enterica serovars Agona and Derby are prevalent among food-producing animals in Chongqing, China
Source: Front Microbiol. 2023 May 25;14:1011719. doi: 10.3389/fmicb.2023.1011719 (PMC10248161; doi:10.3389/fmicb.2023.1011719)
Supplement: Supplementary file 1 [file Table_1.docx]

Supplementary Material

**TABLE S1.** Prevalence of *Salmonella* isolated from different animals

| **Animal** | **Farm** | **No. of samples** | **No. of *Salmonella*** | **Sampling date** |
| --- | --- | --- | --- | --- |
| Pig | Pig farm 1 | 50 | 2 | September 3^rd^, 2016 |
|  | Pig farm 2 | 50 | 1 | January 15^th^,2017 |
|  | Pig farm 3 | 100 | 6 | March 7^th^, 2017 |
|  | Pig farm 4 | 100 | 6 | May 12^th^,2017 |
|  | Pig farm 5 | 50 | 1 | July 2^nd^,2017 |
|  | Pig farm 6 | 50 | 2 | August 31^st^,2017 |
|  | Pig farm 7 | 50 | 4 | October 19^th^,2017 |
|  | Pig farm 8 | 50 | 3 | December 13^th^, 2017 |
|  | Pig farm 9 | 100 | 7 | January 27^th^, 2018 |
|  | Pig farm 10 | 50 | 1 | February 12^th^, 2018 |
|  | Pig farm 11 | 50 | 3 | March 6^th^, 2018 |
|  | Pig farm 12 | 50 | 1 | April 8^th^, 2018 |
|  | Pig farm 13 | 100 | 6 | June 29^th^, 2018 |
|  | Pig farm 14 | 50 | 4 | July 10^th^, 2018 |
|  | Pig farm 15 | 50 | 2 | August 18^th^, 2018 |
|  | Pig farm 16 | 50 | 1 | October 21^st^, 2018 |
|  | Pig farm 17 | 100 | 6 | November 5^th^, 2018 |
|  | Pig farm 18 | 100 | 22 | December 8^th^, 2018 |
|  | Pig farm 19 | 50 | 3 | January 18^th^, 2018 |
|  | Pig farm 20 | 50 | 4 | January 19^th^, 2019 |
|  | Pig farm 21 | 100 | 6 | February 24^th^, 2019 |
|  | Pig farm 22 | 50 | 4 | March 25^th^, 2019 |
|  | Pig farm 23 | 50 | 3 | April 16^th^, 2019 |
|  | Pig farm 24 | 100 | 6 | May 17^th^, 2019 |
|  | **Total** | **1600** | **104** |  |
| Goat | Goat farm 1 | 50 | 1 | October 8^th^, 2016 |
|  | Goat farm 2 | 50 | 1 | February 13^th^, 2017 |
|  | Goat farm 3 | 50 | 2 | June 28^th^, 2017 |
|  | Goat farm 4 | 50 | 1 | November 21^st^, 2017 |
|  | Goat farm 5 | 50 | 1 | January 11^th^, 2018 |
|  | Goat farm 6 | 100 | 3 | October 14^th^, 2018 |
|  | Goat farm 7 | 50 | 2 | April 25^th^, 2019 |
|  | **Total** | **400** | **11** |  |
| Beef cattle | Beef cattle farm 1 | 50 | 1 | July 4^th^, 2017 |
|  | Beef cattle farm 2 | 50 | 2 | September 23^rd^, 2017 |
|  | Total | 100 | 3 |  |
| Rabbit | Rabbit warren 1 | 50 | 2 | September 20^th^, 2017 |
|  | **Total** | **50** | **2** |  |
| Chicken | Chicken farm 1 | 50 | 1 | November 8^th^, 2016 |
|  | Chicken farm 2 | 50 | 2 | April 10^th^, 2017 |
|  | Chicken farm 3 | 50 | 1 | August 9^th^, 2017 |
|  | Chicken farm 4 | 50 | 1 | March 14^th^, 2018 |
|  | Chicken farm 5 | 50 | 1 | May 27^th^, 2018 |
|  | Chicken farm 6 | 50 | 1 | April 22^nd^, 2019 |
|  | **Total** | **300** | **7** |  |
| Duck | Duck farm 1 | 50 | 2 | September 17^th^, 2018 |
|  | **Total** | **50** | **2** |  |
| **Total** |  | **2500** | **129** |  |

**TABLE S2.** Primers used for detection of MLST, β-lactamase, PMQR, and QRDR genes in this study.

| **gene** | **Primer name** | **Primer sequence（5'****~3'）** | **PCR product size (bp)** | **Annealing temperature (℃)** | **References** |
| --- | --- | --- | --- | --- | --- |
| *aroC* | aroC**-**F | CCTGGCACCTCGCGCTATAC | 826 | 60 | General Administration of Quality Supervision, Inspection and Quarantine of the People's Republic of China, 2017 |
|  | aroC**-**R | CCACACACGGATCGTGGCG |  |  |  |
| *dnaN* | dnaN**-**F | ATGAAATTTACCGTTGAACGTGA | 833 | 58 |  |
|  | dnaN**-**R | AATTTCTCATTCGAGAGGATTGC |  |  |  |
| *hemD* | hemD**-**F | GAAGCGTTAGTGAGCCGTCTGCG | 666 | 60 |  |
|  | hemD**-**R | ATCAGCGACCTTAATATCTTGCCA |  |  |  |
| *hisD* | hisD**-**F | GAAACGTTCCATTCCGCGCAGAC | 894 | 60 |  |
|  | hisD**-**R | CTGAACGGTCATCCGTTTCTG |  |  |  |
| *purE* | purE**-**F | ATGTCTTCCCGCAATAATCC | 510 | 58 |  |
|  | purE**-**R | TCATAGCGTCCCCCGCGGATC |  |  |  |
| *sucA* | sucA**-**F | AGCACCGAAGAGAAACGCTG | 643 | 60 |  |
|  | sucA**-**R | GGTTGTTGATAACGATACGTAC |  |  |  |
| *thrA* | thrA**-**F | GTCACGGTGATCGATCCGGT | 852 | 60 |  |
|  | thrA**-**R | CACGATATTGATATTAGCCCG |  |  |  |
| *bla*_TEM_ | TEM-F | CGTGTCGCCCTTATTCCCTTTTTT | 795 | 60 | This study |
|  | TEM-R | TCGTTCATCCATAGTTGCCTGACT |  |  |  |
| *bla*_CTX-M_ | CTX-M-F | ATGTTCGCGGCGGCGGC | 837 | 62 | This study |
|  | CTX-M-R | GGCGATGATTCTCGCCGC |  |  |  |
| *bla*_SHV_ | SHV-F | GGCCGCGTAGGCATGATAGA | 714 | 58 | Chen et al., 2004 |
|  | SHV-R | CCCGGCGATTTGCTGATTTC |  |  |  |
| *bla*_OXA_ | OXA-1-F | ATGAAAAACACAATACATATCAAC | 831 | 50 | This study |
|  | OXA-1-R | TTATAAATTTAGTGTGTTTAGAATGG |  |  |  |
|  | OXA-2-F | ATGGCAATCCGAATCTT | 818 | 50 |  |
|  | OXA-2-R | GCGTCCGAGTTGACTGC |  |  |  |
|  | OXA-10-F | ATGAAAACATTTGCCGCATATG | 801 | 55 |  |
|  | OXA-10-R | TTAGCCACCAATGATGCCC |  |  |  |
| *bla*_CMY_ | CMY-1-F | TGCTGCTGACAGCCTCTTTCT | 1108 | 60 | This study |
|  | CMY-1-R | TTTTCAAGAATGCGCCAGGCC |  |  |  |
|  | CMY-2-F | TCTTTCTCCACGTTTGCCGC | 1043 | 60 |  |
|  | CMY-2-R | TTGTTTGCCAGCATCACGATGC |  |  |  |
|  | CMY-3-F | ATGCAACAACGACAATCCATCCTG | 1149 | 60 |  |
|  | CMY-3-R | TCAACCGGCCAACTGCGC |  |  |  |
| *bla*_PSE_ | PSE-F | AATGGCAATCAGCGCTTCCC | 598 | 58 | Qiao et al., 2017 |
|  | PSE-R | GGGGCTTGATGCTCACTACA |  |  |  |
| *bla*_PER_ | PER-F | ATGAATGTCATCACAAAATG | 927 | 56 |  |
|  | PER-R | TCAATCCGGACTCACT |  |  |  |
| *bla*_VEB_ | VEB-F | CATTTCCCGATGCAAAGCGT | 648 | 55 | Dallenne et al., 2010 |
|  | VEB-R | CGAAGTTTCTTTGGACTCTG |  |  |  |
| *bla*_GES_ | GES-F | AGTCGGCTAGACCGGAAAG | 399 | 55 |  |
|  | GES-R | TTTGTCCGTGCTCAGGAT |  |  |  |
| *qnrA* | qnrA-F | TCAGCAAGAGGATTTCTCA | 627 | 52 | Robicsek et al., 2006 |
|  | qnrA-R | GGCAGCACTATTACTCCCA |  |  |  |
| *qnrB* | qnrB-F | ATGACGCCATTACTGTATAA | 643 | 50 | This study |
|  | qnrB-R | ACGATGCCTGGTAGTTGTCC |  |  |  |
| *qnrC* | qnrC-F | GGGTTGTACATTTATTGAATC | 447 | 50 | Cattoir et al., 2007 |
|  | qnrC-R | TCCACTTTACGAGGTTCT |  |  |  |
| *qnrD* | qnrD-F | CGAGATCAATTTACGGGGAATAG | 596 | 57 | This study |
|  | qnrD-R | ACACCTAAACTCTCAACAAGCTGAA |  |  |  |
| *qnrVC* | qnrVC136-F | AATCAAAGCAATTATATAATCAAGTGAAC | 650 | 55 | This study |
|  | qnrVC136-R | TTAGTCAGGAACAATGATTACCC |  |  |  |
|  | qnrVC457-F | ATGGATAAAACAGACCAGTTATATGTA | 657 | 55 |  |
|  | qnrVC457-R | TTAGTCAGGAACTACTATTAAACCTAAT |  |  |  |
| *qnrS* | qnrS-F | CGTCAACTGCAAGTTCATTGAAC | 435 | 57 | This study |
|  | qnrS-R | TCTAAACCGTCGAGTTCGGC |  |  |  |
| *aac(6′)-Ib-cr* | aac(6′)-Ib-cr-F | CGCAAAAACAAAGTTAGGCATCA | 571 | 57 | This study |
|  | aac(6′)-Ib-cr-R | CTCGAATGCCTGGCGTGTTT |  |  |  |
| *oqxA* | oqxA-F | ATGAGCCTGCAAAAAACCTGG | 1176 | 60 | This study |
|  | oqxA-R | TCAGTTAAGGGTGGCGCTGG |  |  |  |
| *oqxB* | oqxB-F | ATGGACTTTTCCCGCTTTTTTATCGAC | 3153 | 60 | This study |
|  | oqxB -R | CTAGGCGGGCAGATCCTCC |  |  |  |
| *qepA* | qepA-F | GTCTACGCCATGGACCTCAC | 596 | 57 | This study |
|  | qepA-R | AACTGCTTGAGCCCGTAGAT |  |  |  |
| *gyrA* | gyrA-F | CGTTGGTGACGTAATCGGTA | 251 | 60 | Kim et al., 2016 |
|  | gyrA-R | CCGTACCGTCATAGTTATCC |  |  |  |
| *gyrB* | gyrB-F | GTCTGCCGGGCAAACTGGC | 305 | 60 | Hansen et al., 2003 |
|  | gyrB-R | GCCGTCGACGTCCGCATCG |  |  |  |
| *parC* | parC-F | GACGGCCTGAAGCCGGT | 284 | 65 | Kim et al., 2016 |
|  | parC-R | CTCGGCGTATTTGGACAGG |  |  |  |
| *parE* | parE-F | TATCAGGCGATCATGCCGC | 300 | 60 |  |
|  | parE-R | CTTTACCCAAATCGATACGGT |  |  |  |

**Reference**

Cattoir, V., Poirel, L., Rotimi, V., Soussy, C.J., and Nordmann, P. (2007). Multiplex PCR for detection of plasmid-mediated quinolone resistance qnr genes in ESBL-producing enterobacterial isolates. *J. Antimicrob. Chemother.* 60, 394-397. doi: 10.1093/jac/dkm204

Chen, S., Zhao, S., White, D.G., Schroeder, C.M., Lu, R., Yang, H., et al. (2004). Characterization of multiple-antimicrobial-resistant *Salmonella* serovars isolated from retail meats. *Appl. Environ. Microbiol*. 70, 1-7. doi: 10.1128/AEM.70.1.1-7.2004

Dallenne, C., Da Costa, A., Decré, D., Favier, C., and Arlet, G. (2010). Development of a set of multiplex PCR assays for the detection of genes encoding important beta-lactamases in Enterobacteriaceae. *J. Antimicrob. Chemother*. 65, 490-495. doi: 10.1093/jac/dkp498

General Administration of Quality Supervision, Inspection and Quarantine of the People's Republic of China. (2017). Multilocus sequence typing detection method for pathogens in export food―Part 1: *Salmonella*. Industry Standards for Entry-Exit Inspection and Quarantine of the People's Republic of China: No. SN/T 4525.1―2016.

Kim, S.Y., Lee, S.K., Park, M.S., and Na, H.T. (2016). Analysis of the fluoroquinolone antibiotic resistance mechanism of *Salmonella* enterica isolates. *J. Microbiol. Biotechnol.* 26, 1605-1612. doi: 10.4014/jmb.1602.02063

Hansen, H., and Heisig, P. (2003). Topoisomerase IV mutations in quinolone-resistant salmonellae selected *in vitro*. *Microb. Drug. Resist.* 9, 25-32. doi: 10.1089/107662903764736319

Qiao, J., Zhang, Q., Alali, W.Q., Wang, J., Meng, L., Xiao, Y., et al. (2017). Characterization of extended-spectrum β-lactamases (ESBLs)-producing *Salmonella* in retail raw chicken carcasses. *Int. J. Food. Microbiol*. 248, 72-81. doi: 10.1016/j.ijfoodmicro.2017.02.016

Robicsek, A., Strahilevitz, J., Sahm, D.F., Jacoby, G.A., and Hooper, D.C. (2006). *qnr* prevalence in ceftazidime-resistant Enterobacteriaceae isolates from the United States. *Antimicrob. Agents. Chemother.* 50, 2872-2874. doi: 10.1128/AAC.01647-05

**TABLE S3.** Antimicrobial resistance profiles of *Salmonella* isolates recovered from pigs, goats, beef cattle, rabbits, chickens, and ducks as determined by broth dilution method. Unit: %.

| **Antimicrobial** | **Pigs**  **(n=104)** | **Goats**  **(n=11)** | **Beef cattle**  **(n=3)** | **Rabbits**  **(n=2)** | **Chickens**  **(n=7)** | **Ducks**  **(n=2)** | **Total**  **(n=129)** |
| --- | --- | --- | --- | --- | --- | --- | --- |
| Ampicillin | 83 (79.8) | 9 (81.8) | 1 (33.3) | 2 (100.0) | 7 (100.0) | 2 (100.0) | 104 (80.6) |
| Cephalexin | 12 (11.5) | 4 (36.4) | 2 (66.7) | 1 (50.0) | 2 (28.6) | 1 (50.0) | 22 (17.1) |
| Cefazolin | 22 (21.2) | 7 (63.6) | 0 | 0 | 4 (57.1) | 2 (100.0) | 35 (27.1) |
| Cefoxitin | 2 (1.9) | 1 (9.1) | 0 | 0 | 0 | 0 | 3 (2.3) |
| Cefotaxime | 8 (7.7) | 0 | 0 | 0 | 0 | 0 | 8 (6.2) |
| Ceftriaxone | 6 (5.8) | 1 (9.1) | 0 | 0 | 0 | 0 | 7 (5.4) |
| Ceftazidime | 1 (1.0) | 0 | 0 | 0 | 1 (14.3) | 0 | 2 (1.6) |
| Cefepime | 0 | 0 | 0 | 0 | 0 | 0 | 0 |
| Imipenem | 10 (9.6) | 2 (18.2) | 1 (33.3) | 1 (50.0) | 0 | 0 | 14 (10.9) |
| Aztreonam | 7 (6.7) | 1 (9.1) | 0 | 0 | 0 | 0 | 8 (6.2) |
| Streptomycin | 46 (44.2) | 5 (45.5) | 0 | 1 (50.0) | 3 (42.9) | 2 (100.0) | 57 (44.2) |
| Kanamycin | 28 (26.9) | 4 (36.4) | 1 (33.3) | 2 (100.0) | 4 (57.1) | 0 | 39 (30.2) |
| Gentamicin | 36 (34.6) | 2 (18.2) | 1 (33.3) | 2 (100.0) | 4 (57.1) | 1 (50.0) | 46 (35.7) |
| Amikacin | 1 (1.0) | 0 | 0 | 0 | 0 | 0 | 1 (0.8) |
| Tetracycline | 86 (82.7) | 8 (72.7) | 0 | 2 (100.0) | 5 (71.4) | 2 (100.0) | 103 (79.8) |
| Doxycycline | 95 (91.3) | 8 (72.7) | 1 (33.3) | 2 (100.0) | 5 (71.4) | 2 (100.0) | 113 (87.6) |
| Chloramphenicol | 78 (75.0) | 6 (54.5) | 0 | 2 (100.0) | 6 (85.7) | 2 (100.0) | 94 (72.9) |
| Florfenicol | 80 (76.9) | 8 (72.7) | 1 (33.3) | 2 (100.0) | 6 (85.7) | 2 (100.0) | 99 (76.7) |
| Nalidixic acid | 26 (25.0) | 3 (27.3) | 1 (33.3) | 2 (100.0) | 4 (57.1) | 2 (100.0) | 38 (29.5) |
| Norfloxacin | 6 (5.8) | 1 (9.1) | 0 | 2 (100.0) | 2 (28.6) | 0 | 11 (8.5) |
| Ciprofloxacin | 28 (26.9) | 3 (27.3) | 0 | 2 (100.0) | 5 (71.4) | 1 (50.0) | 39 (30.2) |
| Ofloxacin | 2 (1.9) | 1 (9.1) | 0 | 2 (100.0) | 2 (28.6) | 0 | 7 (5.4) |
| Enoxacin | 4 (3.8) | 1 (9.1) | 0 | 2 (100.0) | 1 (14.3) | 0 | 8 (6.2) |
| Gatifloxacin | 2 (1.9) | 1 (9.1) | 0 | 0 | 1 (14.3) | 0 | 4 (3.1) |
| Trimethoprim-sulfamethoxazole | 74 (71.2) | 8 (72.7) | 1 (33.3) | 2 (100.0) | 5 (71.4) | 2 (100.0) | 92 (71.3) |
| Trimethoprim | 80 (76.9) | 8 (72.7) | 1 (33.3) | 2 (100.0) | 7 (100.0) | 2 (100.0) | 100 (77.5) |

**TABLE S4.** The prevalence of β-lactamase genotype forms in *Salmonella* isolates originating from different animals.

| **Genotype form of β-lactamase genes** | **Pigs (%)** | **Goats (%)** | **Beef cattle (%)** | **Rabbits (%)** | **Chickens (%)** | **Ducks (%)** | **Total (%)** |
| --- | --- | --- | --- | --- | --- | --- | --- |
| TEM-1 | 29 (27.9) | 5 (45.5) | 1 (33.3) |  | 4 (57.1) | 1 (50.0) | 40 (31.0) |
| TEM-1, OXA-1 | 1 (1.0) |  |  |  | 1 (14.3) |  | 2 (1.6) |
| TEM-1, OXA-10 | 5 (4.8) |  |  |  |  |  | 5 (3.9) |
| TEM-1, OXA-10, CMY-116 | 1 (1.0) |  |  |  |  |  | 1 (0.8) |
| TEM-1, CMY-116 |  |  |  |  |  | 1 (50.0) | 1 (0.8) |
| TEM-1, CTX-M-65 | 8 (7.7) |  |  |  |  |  | 8 (6.2) |
| TEM-1a | 15 (14.4) |  |  |  |  |  | 15 (11.6) |
| TEM-1a, CMY-116 | 1 (1.0) |  |  |  |  |  | 1 (0.8) |
| TEM-1b | 10 (9.6) |  |  |  |  |  | 10 (7.8) |
| TEM-116 | 1 (1.0) |  |  |  |  |  | 1 (0.8) |
| TEM-171 | 11 (10.6) | 1 (9.1) | 1 (33.3) |  | 1 (14.3) |  | 14 (10.9) |
| TEM-171, OXA-1 | 6 (5.8) | 2 (18.2) |  | 1 (50.0) |  |  | 9 (7.0) |
| OXA-1 | 6 (5.8) | 1 (9.1) |  | 1 (50.0) | 1 (14.3) |  | 9 (7.0) |

**TABLE S5.** The prevalence of PMQR genotype forms in *Salmonella* isolates originating from different animals.

| **Genotype form of PMQR genes** | **Pigs (%)** | **Goats (%)** | **Rabbits (%)** | **Chickens (%)** | **Ducks (%)** | **Total (%)** |
| --- | --- | --- | --- | --- | --- | --- |
| *qnrB6*, *oqxA1*, *oqxB5*, *aac(6')-Ib-cr* | 5 (4.8) |  |  |  |  | 5 (3.9) |
| *qnrB6*, *oqxB5*, *aac(6')-Ib-cr* | 1 (1.0) |  |  |  |  | 1 (0.8) |
| *qnrB6*, *aac(6')-Ib-cr* | 5 (4.8) |  |  |  |  | 5 (3.9) |
| *qnrD1*, *qnrS10*, *oqxA1*, *oqxB5* | 1 (1.0) |  |  |  |  | 1 (0.8) |
| *qnrD1*, *oqxA1*, *oqxB5*, *aac(6')-Ib-cr* | 1 (1.0) |  |  |  |  | 1 (0.8) |
| *qnrS1* | 2 (1.9) | 2 (18.2) |  |  |  | 4 (3.1) |
| *qnrS1*, *oqxB5* | 1 (1.0) |  |  |  |  | 1 (0.8) |
| *qnrS2* | 1 (1.0) |  |  |  |  | 1 (0.8) |
| *qnrS2*, *oqxA1*, *oqxB5*, *aac(6')-Ib-cr* | 5 (4.8) | 2 (18.2) | 1 (50.0) |  |  | 8 (6.2) |
| *qnrS2*, *aac(6')-Ib-cr* | 1 (1.0) | 1 (9.1) |  |  |  | 2 (1.6) |
| *qnrS10* | 12 (11.5) | 1 (9.1) |  | 1 (14.3) |  | 14 (10.9) |
| *qnrS10*, *oqxA1*, *oqxB5* | 1 (1.0) |  |  |  |  | 1 (0.8) |
| *qnrS10*, *oqxB5* | 2 (1.9) |  |  |  |  | 2 (1.6) |
| *oqxA1* | 1 (1.0) |  |  |  |  | 1 (0.8) |
| *oqxA1*, *oqxB5* | 10 (9.6) |  |  |  |  | 10 (7.8) |
| *oqxA1*, *oqxB5*, *aac(6')-Ib-cr* | 4 (3.8) |  |  | 1 (14.3) |  | 5 (3.9) |
| *oqxA1*, *aac(6')-Ib-cr* |  |  | 1 (50.0) | 1 (14.3) |  | 2 (1.6) |
| *oqxB5* | 6 (5.8) |  |  |  |  | 6 (4.7) |
| *oqxB5*, *aac(6')-Ib-cr* | 2 (1.9) |  |  |  |  | 2 (1.6) |
| *aac(6')-Ib-cr* | 7 (6.7) |  |  | 2 (28.6) | 1 (50.0) | 10 (7.8) |
